# Reply to: Adequacy of enteral nutrition support in intensive care units does not affect the short- and long-term prognosis of mechanically ventilated patients: a pilot study

**DOI:** 10.5935/0103-507X.20200101

**Published:** 2020

**Authors:** Cecília Flávia Lopes Couto, Ângela Dariano, Cassiano Texeira, Carolina Hauber da Silva, Anelise Bertotti Torbes, Gilberto Friedman

**Affiliations:** 1 Postgraduate Program in Pneumological Sciences, Universidade Federal do Rio Grande do Sul - Porto Alegre (RS), Brazil.; 2 Intensive Care Unit, Hospital Moinhos de Vento - Porto Alegre (RS), Brazil.; 3 Santa Casa de Misericórdia de Porto Alegre, Universidade Federal de Ciências da Saúde de Porto Alegre - Porto Alegre (RS), Brazil.

**To the Editor**

In our study “Adequacy of enteral nutrition support in intensive care units does not affect the short- and long-term prognosis of mechanically ventilated patients: a pilot study”^(1)^ the sample size was calculated to assess the short-term effects, and in this sense, it is not a pilot study. However, we had no information available in the literature, much less in the Brazilian settings about what the sample size would be for a long-term study. Thus, it was considered a pilot study, which allowed us to estimate the magnitude of losses and deaths and to calculate the sample.^(2)^

The absence of data about what was effectively offered is the limitation. The patients were separated into two different groups (≥ 70% *versus* < 70%) of calorie intake adequacy considering only the records of what was prescribed, which constitutes information that can be recovered in medical records. In the hospital where the study was conducted, unfortunately, there is no record of what was effectively offered or, better yet, what was delivered. This is a common limitation in this type of study. The progression of the enteral diet is registered in medical records, but sometimes the offer is less (for example pause for a tomography).

The lack of evaluation of the functional capacity at the intensive care unit (ICU) admission is an important limitation. Even so, it is reasonable to speculate that patients who recover over time do so by nutritional adequacy or by tolerance to diet progression - the latter indicating that they would be less severe already in the ICU.

The inference is speculative. The sample size does not allow definitive conclusions, and the article makes this clear from the title. The title (“a pilot study”) is a clear message that the information will eventually be used in planning a larger study.

We appreciate the considerations and take them all as pertinent. We hope we have clarified the main ones.
